# Condom use and associated factors among men who have sex with men in Togo, West Africa

**DOI:** 10.11604/pamj.2016.23.118.7492

**Published:** 2016-03-24

**Authors:** Tchaa Abalo Bakai, Didier Koumavi Ekouevi, Boris Kévin Tchounga, Eric Balestre, Kossivi Agbélénko Afanvi, Kariyiare Benjamin Goilibe, Yao Kassankogno, Vincent Palokinam Pitche

**Affiliations:** 1 Université de Lomé, Département de Santé Publique, Faculté des Sciences de la Santé, Lomé, Togo; 2 Centre Africain de Recherche en Epidémiologie et Sante Publique (CARESP), Lomé, Togo; 3 Université de Bordeaux, ISPED, Centre INSERM U897- Epidémiologie-Biostatistique, Bordeaux, France; 4 Inserm U897, ISPED, Université de Bordeaux, Bordeaux France; 5 Programme PAC-CI, CHU de Treichville, Abidjan, Côte d'Ivoire; 6 Programme National de Lutte contre le VIH/Sida, Lomé, Togo; 7 CHU Sylvanus Olympio, Service de Dermatologie et Infectiologie, Lomé, Togo

**Keywords:** HIV prevalence, MSM, condom use, Togo

## Abstract

**Introduction:**

In 2011, the prevalence of HIV among men who have sex with men (MSM) in Togo was estimated at 19.6% compared to 3.4% in the general population. This study aimed to describe condom use and associated factors among MSM in Togo.

**Methods:**

In 2011, a cross-sectional survey was conducted using the snowball sampling method among MSM in Togo. This study enrolled MSM aged 18 years and above who reported having sexual contact with other men within the last 30 days. A standardized survey form was used for data collection, and multivariate analyses were performed.

**Results:**

A total of 724 MSM were included in this study. The median age was 25 years [22-28], 90.3% had at least a secondary school level. The sexual practices during the last sexual encounter with another man included: insertive anal sex (62.2%), receptive anal sex (56.6%), oral sex (33.8%) and oral-anal sex (8.6%). A condom was used during the last insertive and receptive anal encounters in 78.4% and 81.2% of the time, respectively. In multivariate analysis, condom use was positively associated with previous participation in HIV/STD prevention activities (aOR=1.72; 95% CI=[1.09-2.71]), with the consideration of the last sexual partner as a casual one (aOR=1.87; 95% CI=[1.24-2.82]) and with having at least a secondary school level (aOR=2.40; 95% CI=[1.22-4.69]).

**Conclusion:**

One out of five MSM did not use a condom during the last anal encounter with another man. HIV prevention programs in Africa should develop specific interventions targeting MSM to reduce the incidence of HIV in this hidden population.

## Introduction

The prevalence of HIV infection among men who have sex with men (MSM) is higher than in the general population worldwide [1, 2]. It is estimated that only one MSM out of ten has access to HIV preventive care, and this proportion is lower in resource-constrained settings [3]. Moreover, in sub-Saharan Africa, communication strategies for HIV prevention focus primarily on heterosexual transmission leading to the misconception among MSM that same-sex intercourse has a lower risk of HIV transmission [4]. However, many studies conducted in Senegal and other sub-Saharan African countries reported higher HIV prevalence among MSM (2 to 20 times) than in the overall population [2, 5–8]. This large difference in HIV prevalence could be explained by the route of transmission. Indeed, the per act probability of HIV transmission during anal intercourse is 17 to 18 times higher than that during vaginal intercourse [9]. Other factors, such as the stigmatization and discrimination against MSM [10–12], the difficult access to prevention services [2, 13], the multiplicity of sexual partners and the inconsistent condom use [2] could also play a role. In 2007, an epidemiological and behavioral survey conducted among MSM in Senegal (enquêteELIHoS) reported a high frequency of unsafe anal intercourse (UAI) with only one out of four MSM reporting having used a condom during the last sexual contact with another man [14]. In Togo, the HIV prevalence among MSM was estimated at 19.6% in 2011 [15], which is six times higher than the estimated 3.4% national prevalence [16]. This prevalence varies between Lomé, the capital of Togo, (29.6%) and other cities (average of 4.6%). The same study identified a low level of knowledge regarding HIV transmission and prevention [15]. Moreover, no survey on sexual behavior and condom use among MSM has been conducted in Togo to date. This study aimed to describe condom use and associated factors among MSM in Togo.

## Methods

### Study design

This study was part of a sero-prevalence survey conducted among MSM in six cities in Togo from November 2011 to January 2012. It was designed and coordinated by a working group that included community physicians, epidemiologists, social workers and MSM community leaders. The objectives of this survey were to estimate the sero-prevalence of HIV infection among MSM, to evaluate their knowledge, attitudes and practices regarding HIV and STD prevention, to evaluate their access to health facilities and finally to describe the sexual behavior, condom use and associated factors among MSM in Togo [15]. The current analyses described condom use and associated factors during the last same-sex encounter among MSM in Togo.

### Study population

The study population was composed of MSM aged 18 years and above who had been living in Togo for at least three months. They were recruited in the six largest cities of Togo (Lomé, Aného, Tsévié, Kpalimé, Sokodé and Kara), which are the regional capitals of the six regions of the country [15]. The snowball sampling method was used to recruit participants. First, MSM community leaders were identified and recruited for the study through the national non-governmental organization (NGO) ”Arc en Ciel„. This NGO, which is familiar with research activities, identified the MSM community leaders in the local and regional MSM associations based on their previous experience with research projects. Overall, 17 MSM leaders were recruited based on the population size of each region: one MSM leader was recruited for each town with fewer than 100,000 inhabitants (Aného, Tsévié, Kpalimé, Sokodé and Kara), and 12 were recruited in Lomé, the capital. All of the community leaders received theoretical and practical training in conducting interviews with a survey form. Each of the community leaders was expected to recruit at least 30 MSM in his network and region.

### Data collection

A structured and standardized survey form adapted from the form used in Senegal in 2007 was administered to each participant by the MSM community leaders. The survey form collected data on the patient's demographics, social life, knowledge, attitude and practices regarding HIV/STDs, condom use and sexual behavior. To avoid stigmatization, the survey form was administered at a convenient location selected by each participant.

### Data analysis

The data from the survey forms were entered using Epi Data software version 3.1 (The Epi Data Association, Odense, Denmark), and Stata^®^ software version 9.0 (Stata Corp, College Station, Texas, USA) was used for analyses. Pearson's Chi square and Fisher's exact tests were used to compare proportions. Condom use during the last anal encounter was the dependent variable, and we first performed a univariate analysis considering the following group of variables as independent variables: demographic characteristics, sexual behavior, HIV testing, participation in prevention activities and membership in MSM associations. We then performed a multivariate analysis including the variables associated with the dependent variable with p-values <0.25. The multivariable logistic regression analysis was performed using a backward stepwise procedure, and the fit of the final model was verified using the Hosmer-Lemeshow test.

### Ethics statement

This study was conducted with the approval of the national bioethics committee for research in Togo. In addition, each participant gave a written consent before being included in the study.

## Results

**Figure 1:**
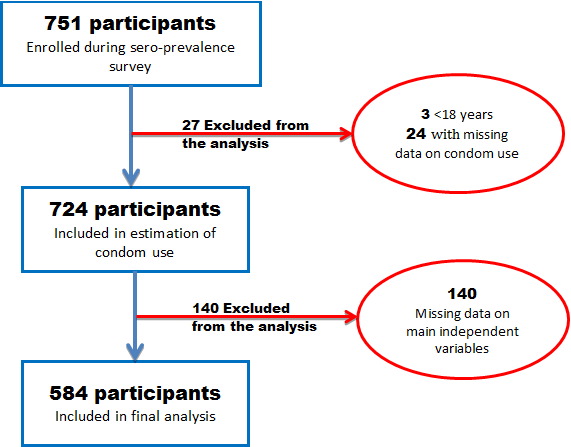
Study population flowchart

Overall, 751 MSM participated in this study; 27 were excluded from the current analysis due to age < 18 years or to missing data on condom use. Thus, the data on 724 MSM were considered to describe condom use in this population. For the analysis of factors associated with condom use, 140 survey forms were excluded due to important missing data, especially for the independent variables. However, the participants excluded (n=140) and those considered in this analysis (n=584) were not significantly different with regard to demographic characteristics (p-value ≥ 0.05) (Table 1). Figure 1 presents the selection flow chart of the population considered for this analysis.

**Table 1:** Demographic characteristics of MSM included and excluded from the main analyses

	Included (N=584)	Excluded (N=140)	Total (N=724)	P-value*
***Age of MSM participant (years)***	**N (%)**	**N (%)**	**N (%)**	**0.706**
<25	310 (53.1)	73 (52.1)	383(52.9)	
≥25	274 (46.9)	60 (42.8)	334 (39.2)	
Missing	0 (0.0)	7 (5.1)	7(0.9)	
***Age of most recent male partner (years)***				0.864
<25	270 (46.2)	38 (27.1)	308 (42.5)	
≥25	314 (53.8)	46 (32.8)	360 (49.8)	
Missing	0 (0.0)	56 (40.1)	56 (7.7)	
***Membership to MSM association***				0.313
No	478 (81.8)	76 (54.3)	554 (76.5)	
Yes	106 (18.2)	22 (15.7)	128 (17.7)	
Missing	0 (0.0)	42 (30.0)	42 (5.8)	
***Location of last sexual encounter***				0.452
Home	519 (88.9)	116 (82.8)	635 (87.7)	
Public place	65 (11.1)	18 (12.9)	83 (11.5)	
Missing	0 (0.0)	6 (4.3)	6 (0.8)	
***Relationship with most recent male partner***				0.695
Occasional partner	268 (45.9)	66 (47.1)	334 (46.1)	
Regular partner	316 (54.1)	52 (37.2)	368 (50.9)	
Missing	0 (0.0)	22 (15.7)	22 (3.0)	
***Participation in HIV prevention activities***				0.414
No	306 (52.4)	59 (42.1)	365 (50.4)	
Yes	278 (47.6)	45 (32.2)	323 (44.7)	
Missing	0 (0.0)	36 (25.7)	36 (4.9)	
***Screened for HIV***				0.061
No	206 (35.3)	56 (40.0)	262 (36.2)	
Yes	378 (64.7)	68 (48.6)	446 (61.6)	
Missing	0 (0.0)	16 (11.4)	16 (2.2)	
***In love with most recent male partner***				0.968
No	224 (38.4)	50 (35.7)	274 (37.8)	
Yes	360 (61.6)	81 (57.9)	441 (61.0)	
Missing	0 (0.0)	9 (6.4)	9 (1.2)	
***Aware of recent male partner's HIV status***				0.585
No	453 (77.4)	101 (72.1)	554 (76.5)	
Yes	131 (22.4)	33 (23.6)	164 (22.7)	
Missing	0 (0.0)	6 (4.3)	6 (0.8)	
***Education level***				0.240
Primary school	45 (7.7)	14 (10.0)	59 (8.1)	
Secondary school or higher	539 (92.3)	115 (82.2)	654 (90.4)	
Missing	0 (0.0)	11 (7.8)	11 (1.5)	
***Marital status***				0.435
Single	472 (80.8)	98 (70.0)	570 (78.7)	
Married/being committed	112 (19.2)	21 (15.0)	133 (18.4)	
Missing data	0 (0.0)	21 (15.0)	21 (2.9)	
***Living with a partner***				0.237
No	448 (76.7)	97 (69.3)	545 (75.3)	
Yes with a man	81 (13.9)	11 (7.8)	92 (12.7)	
Yes with a woman	55 (9.4)	7 (5.0)	62 (8.6)	
Missing data	0 (0.0)	25 (17.9)	25 (3.4)	
***Total***	584 (100.0)	140 (100.0)	724 (100.0)	

* These p-values were computed ignoring missing values for both groups

### Demographic characteristics of the MSM population

Most of the participants enrolled in the study (91.0%) were Togolese, had a median age of 25 years [interquartile range (IQR) 22-28] and reported having a secondary school educational level (90.4%). The majority (78.7%) were unmarried, 92 others (12.7%) were living with another man and 62 (8.6%) were married and living with a woman. Only 128 participants (17.7%) were members of an MSM association (Table 1).

### Condom use and sexual behavior among MSM

**Figure 2:**
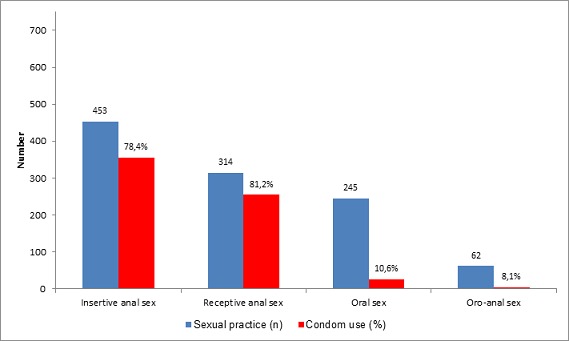
Condom use according to sexual practices among MSM in Togo

The frequency of condom use varied according to sexual practices: 78.4% during insertive anal sex, 81.2% during receptive anal sex and less than 10% during oral sex (Figure 2). Nearly 42.5% of the participants had sexual partners aged less than 25 years, and 11.5% reported having had their last sexual intercourse in a public place. In addition, 76.6% declared that they were unaware of the HIV serological status of their last partner, and 50.4% had never attended HIV/STD prevention activities (Table 1).

### Factors associated with condom use

Table 2 shows the results of the logistic regression analyses. In the univariate analysis, condom use was positively associated with having already had an HIV test (OR=1.54; 95% CI= [1.04-2.29], p=0.03) or considering the last sexual partner as a casual partner (OR=1.76; 95% CI =[1.19-2.61], p < 0.01). Moreover, taking part in HIV/STD prevention activities (OR=2.20; 95% CI=[1.47-3.31], p < 0.01), having at least a secondary school educational level (OR=2.21; 95% CI= [1.16-4.19], p=0.02) and being a member of an MSM association (OR=2.42; 95% CI= [1.31-4.49], p < 0.01) were positively associated with condom use. In the multivariate analysis, condom use remained positively associated with previous participation in HIV/STD prevention activities (aOR=1.72; 95% CI=[1.09-2.71], p=0.02), with having considered the last sexual partner to be a casual one (aOR=1.87; 95% CI=[1.24-2.82], p < 0.01) and with having at least a secondary school educational level (aOR=2.40; 95% CI=[1.22-4.69] p=0.01).

**Table 2:** Factors associated with condom use during the last anal intercourse among MSM in Togo in 2012, uni and multivariable analyses

	Univariate analysis			Multivariate analysis (final model)		
	OR	IC 95%	P-value	AOR*	IC 95%	P-value
*Age of MSM participant (years)*						
**≥25/ <25**	1.37	0.92-2.03	0.121	-	-	-
						
*Age of most recent male partner (years)*						
**≥25/ <25**	1.03	0.70-1.53	0.861	-	-	-
						
*Membership to MSM association*						
**Yes/ No**	2.42	1.31-4.49	**0.005**	-	-	**-**
						
*Location of last sexual encounter*						
**Public/ Home**	1.45	0.73-2.87	0.289	-	-	-
						
*Relationship with most recent male partner*						
**Casual** / **Regular**	1.76	1.19-2.61	**0.004**	1.87	1.24-2.82	**0.003**
						
*Participation in HIV/STD prevention activities*						
**Yes/ No**	2.20	1.47-3.31	**0.001**	1.72	1.09-2.71	**0.019**
						
*Having had HIV testing*						
**Yes/ No**	1.54	1.04-2.29	**0.033**	-	-	-
						
*Being in love with the last partner*						
**Yes/ No**	1.25	0.84-1.87	0.264	-	-	
						
*Aware of most recent male partner's HIV status*						
**Yes/ No**	1.13	0.70-1.81	*0.624*	-	-	
						
*School level*						
**Secondary school or higher / Primary school**	2.21	1.16-4.19	**0.015**	2.40	1.22-4.69	**0.010**

* AOR: Adjusted Odds Ratio

## Discussion

This study reveals that three out of four MSM used a condom during their last anal intercourse in Togo. Condom use was positively associated with a higher educational level, participation in HIV/STD prevention activities and having sex with a casual partner. Condom use is one of the most effective strategies to prevent sexual transmission of HIV in both MSM and heterosexual populations [17, 18]. In Togo, condoms were used during the last insertive and receptive anal intercourses by 78.4% and 81.2% of MSM, respectively. Although these rates seem higher than those reported in Cameroon (43% in the past 6 months) [19], Senegal (77% for insertive or receptive anal sex) [14], Egypt (19% at the last encounter) [20] and Jordan (27% at the last encounter) [21], the comparison is difficult because most of these surveys assessed not only condom use during the last anal encounter but also the consistency of condom use in the previous month or the last six months. In addition, other factors such as the main religion in the country, the prevalence of HIV and the inclusion of key populations in the HIV prevention strategies could influence the condom use in the different countries. However, these results suggest that 20% to 25% of MSM did not use a condom when having anal sex. This result is particularly worrisome considering the high prevalence of HIV infection among MSM in Togo (19.6% at the time of the study) [15]. Indeed, it has been previously demonstrated that the risk of HIV transmission for receptive and insertive anal intercourse (138/10000, and 11/10000 respectively) is higher than the risk associated with penile-vaginal and oral-genital intercourse [9]. Thus, the fact that more than one MSM out of five did not use a condom during the previous anal encounter could explain the high HIV prevalence in this specific group. It is thus imperative to significantly improve condom use among MSM in Togo to mitigate HIV transmission and reduce HIV incidence. The last decade in Togo has been characterized by a decreasing HIV prevalence in the general population due to a decrease in HIV transmission [22]. This change is the result of prevention activities emphasizing condom use and targeting young and sexually active populations [16]. Similar activities targeting MSM, are still rare in Togo, likely due to the stigmatization and penalization of homosexuality. This study reveals a significant link between participation in previous HIV/STD prevention activities and condom use during the last anal intercourse. This result emphasizes the need to implement HIV/STD prevention activities specifically targeting MSM with a message designed to address MSM sexual behaviors.

Moreover, MSM in Africa have limited access to government health facilities due to stigmatization; they often rely on MSM associations that provide healthcare and address MSM health concerns. As MSM usually meet each other and discuss their problems within these organizations, they could constitute an interesting entry point for public health interventions targeting this hidden population. In the univariate analysis, our study revealed a significant relation between MSM association's membership and condom use, but this association was not significant in multivariate analysis. This result suggests that being member of an MSM association only is not sufficient to improve condom use among MSM; it is also necessary for MSM to take part in HIV/STD prevention activities. In Senegal in 2007, the frequency of unsafe anal sex was three times higher among participants who did not belong to an MSM association [14] and the main reason was the fact that the prevention activities and special follow up were organized for MSM in the associations. It is therefore critical for the MSM associations to take the lead in HIV prevention among MSM populations by relaying prevention messages and implementing other prevention strategies targeting this specific population. However, to achieve this goal, MSM associations need increased capacity and more funding to provide MSM with a substantial package of specific health care. Overall, condom use is less frequent with a regular partner (or one with whom there is an emotional attachment) in both homosexual and heterosexual populations [14, 19]. Like other reports from Cameroon [19] and Senegal [14], our study found a low proportion of condom use with regular partners or partners with whom the participants reported being in love. However, considering the high HIV prevalence in this setting and the high proportion of MSM ignoring the HIV status of their last partner, condom use should be systematically recommended even with a regular sexual partner, as it has been reported that 68% of HIV transmissions among MSM were from main partners and could be attributed to lower condom use with this main partner [23]. Once again, the role of MSM associations will be critical in relaying information, conducting HIV screening and delivering results in conventional hospital or VCT settings and referring MSM to HIV care programs.

The main limitation of this study is the use of the snowball sampling method, which results in a selection bias [24]. However, in Togo, where homosexuality is still penalized, it is very difficult to reach this hidden population. Thus, it was easier to use snowball sampling method that rely on MSM community leaders. Moreover, the use of MSM community leaders has probably increased the social desirability bias, which leads to an over declaration of condom use. Another limitation is the exclusion of participants with missing data for the main independent variables in the model. After comparing the included and excluded participants, there was no significant difference between the two groups. Another concern is that we did not collect data measuring stigma or healthcare access during this survey; these two factors could be related to condom access and could influence condom use. Finally, the questionnaire used did not allow collecting data on the time frame and consistency intake of some important variables such as ”attending HIV/STD prevention activities„, we were therefore unable to explore how these measurements may have impacted the condom use. Nevertheless, this is one of the largest surveys on MSM sexual behavior in sub-Saharan Africa with more than 700 participants recruited throughout the six main cities of the six regions of Togo. This study describes condom use and sexual practices among MSM and highlights the positive role of health promotion activities and community associations as an entry point in the fight against HIV and other STDs. The lessons learned from this study could help AIDS control programs design specific activities and integrated strategies for the reduction of HIV incidence among MSM in Togo. These interventions could be implemented through associations to reach as many MSM as possible. However, there is a need for specific studies to evaluate the effectiveness of community-based strategies to deliver HIV prevention services to MSM in the context of stigmatization.

## Conclusion

This study reports insufficient condom use among MSM in Togo, particularly during the most recent anal intercourse. African AIDS control programs should develop specific MSM-oriented interventions for the promotion of condom use.

### What is known about this topic

- HIV prevalence is higher among MSM than in the general population, either in developing and developed countries.
- In Togo, HIV prevalence among MSM is 19%, six times the national HIV prevalence and due to discrimination and stigma, most MSM also have sexual intercourse with women (girlfriend or spouse).
- MSM in Togo have a very low level of knowledge regarding HIV transmission and prevention, this could partly explain the high transmission rate in this population.

### What this study adds

- This study adds additional data on condom used among MSM in sub-Saharan Africa and shows that condom use is high among MSM in Togo, although 25% of them still have unsafe anal intercourses.
- This study underlines the factors associated with consistent condom use among MSM in Togo.
- This study suggests new approach and guidance for policy makers and caregivers to improve condom use among MSM.
